# Disrespect and Abuse during Childbirth in Ethiopia: A Systematic Review

**DOI:** 10.1155/2020/8186070

**Published:** 2020-10-23

**Authors:** Meresa Berwo Mengesha, Asgele Gebrekrstos Desta, Hayat Maeruf, Hagos Degefa Hidru

**Affiliations:** ^1^Adigrat University College of Medicine and Health Sciences, Department of Clinical Midwifery, Adigrat, Tigray, Ethiopia; ^2^Adigrat University College of Medicine and Health Science, Department of Pediatrics and Child Health Nursing, Adigrat, Tigray, Ethiopia; ^3^Adigrat University College of Medicine and Health Sciences, Department of Public Health, Institute of Epidemiology, Adigrat, Tigray, Ethiopia

## Abstract

**Background:**

Disrespect and abuse are recognized for the restricting impact of women from seeking maternal care, psychological humiliations, grievances, and unspoken sufferings on women during childbirth. Individual primary studies are limited in explaining of extent of disrespect and abusive care. Hence, this review considers the synthesis of comprehensive evidence on the extent, contributing factors, and consequences of disrespectful and abusive intrapartum care from the women's and providers' perspectives in Ethiopia.

**Methods:**

Articles had been systematically searched from the databases of PubMed, Cochrane Library, POPLINE, Google Scholar, HINARI, African Journals Online, and WHO Global Health Library. A qualitative and quantitative synthesis was performed using the Bowser and Hill landscape analytical framework.

**Result:**

Twenty-two studies comprised of the 16 quantitative; 5 qualitative and one mixed studies were included. The most repeatedly dishonored right during facility-based childbirth in Ethiopia was nondignified care, and the least commonly reported abuse was detention in health facilities. These behaviors were contributed by normalization of care, lack of empowerment and education of women, weak health system, and lack of training of providers. Women subjected to disrespectful and abusive behavior distanced themselves from the use of facility-based childbirth-related services and have endured psychological humiliations.

**Conclusion:**

Disrespectful and abusive care of women during childbirth is repeatedly practiced care in Ethiopia. This result specifically described the contributing factors and their effects as a barrier to the utilization of facility-based childbirth. Therefore, to overcome this alarming problem, health systems and care providers must be responsive to the specific needs of women during childbirth, and implementing policies for standard care of respectful maternity care must be compulsory. In addition, observational, qualitative, and mixed types of studies are required to provide comprehensive evidences on disrespect and abusive behavior during childbirth in Ethiopia.

## 1. Background

Respectful maternity care during childbirth has been called, “care coordinate and given to all women in a manner that maintains their dignity, privacy and confidentiality, ensures freedom from harm and mistreatment, and allows informed choice and continuous support during labour and childbirth”[1]. In comparison to respectful care, disrespect and abusive (D and A) care during childbirth suggests a divergence from the right to health and is an indication of the standard of childbirth services [2].

It is widely acknowledged at the policy level that all women should have the right to respectful, dignified care during labour, and childbirth [3]. Obstetric violence and mistreatment during childbirth is a global problem, however, its worst form is in low-income countries such as Sub-Saharan Africa [2, 4–10]. D and A, as a global epidemic, puts a women into a grievance, unspeakable suffering, psychological embarrassment, prohibits women from accessing maternity services from health institutions, may not plan to come health institutions in the coming future, and influence women's decision about how, when, and with whom to give birth [11–14].

Ethiopia, one of the world's major contributors to maternal mortality in Sub-Saharan African countries, has recorded 412 maternal deaths per 100,000 live births [15]. Just 48% of mothers are attended by skilled birth personnel in Ethiopia [16]. Despite a lot of effort deemed to promote health services in Ethiopia, low-quality services continue to be a challenge [17–19]. Just 29.2 percent of mothers received standardized care during childbirth, with little to no quality of services are attributed by few skilled personnel, lack of resources, and lack of transportation services in the event of emergency, if any [20]. In addition to inadequate access to facility-based childbirth, low utilization of skilled birth attendance during childbirth is augmented by abusive, mistreatment, and humiliation of care providers after they reached health facilities that are easily jeopardized by poor quality of service [4, 11, 21] but skilled birth services are considered to be a critical stratagem in reducing maternal mortality. To implement the sustainable development goals (SDGs) aimed at reducing maternal mortality ratio to 70 per 100000 live births [16, 22]. It is difficult to execute this ambitious plan without promoting respectful maternal care (RMC) which has been recognized as an essential component of strategies to improve utilization and quality of maternity care [3].

Disrespect and abusive care in its wider term composed attitudes and actions of health care providers, health policy and institutional failure, deviations from national expectation of good quality care, or deviations from human rights standards [23]. D and A can be categorized into six domains: physical abuse, lack of privacy and confidentiality, unconsented care, non- dignified care, discrimination, abandonment of care and detention in health facilities [24].

Studies on the psychosocial component of childbirth care are minimal, based primarily on the professional efficiency care provider and women's satisfaction, and perhaps the attitude and conduct of the health care provider is one of the deterrents to the quality of intrapartum care [2, 21, 25].

While many women suffer from disrespect and abusive care during childbirth, such experiences are not well recorded and taken into account factors in the planning of maternal health services [2]. There are few awareness and mitigating initiatives in place in Ethiopia to promote respectful maternity care. The government of Ethiopia in its five-year progressing plan, called the Health sector transformation plan, personalized a compassionate respectful care initiative, offering sector-wide training for health care providers including addressing the issue of respectful maternity care [19, 26]. While such initiatives are considered to be in a position to mitigate this alarming problem, their considerations in the planning of maternal health services and the implementation of concrete actions are not promising in the national context. This review would therefore allow policymakers, program designers, and interested partners in this field to apprehend the comprehensive extent of D and A in Ethiopia.

To the best of our knowledge, only one similar review was done in Ethiopia [27] which found that the overall pooled prevalence of D and A during childbirth and maternity care in Ethiopia was 49.4% included the synthesis of only seven studies. The review focused on quantitative synthesis (pooled prevalence of type of disrespect and abuse only). The previous review did not include qualitative studies, many of the studies done were quantitative, did not give emphasis on providers perspectives, did not explore contributing factors and consequences of disrespectful and abusive care were not identified. Hence, this review is aimed at synthesizing comprehensive evidence on the nature and extent of D and A, contributing factors, and consequences of disrespectful and abusive intrapartum care from the women's and providers' perspectives and to forward recommendations in Ethiopia.

## 2. Methods

### 2.1. Data Sources and Search Strategies

This systematic review was prepared in line with the recommendation of Preferred Reporting Items for Systematic Review and Meta-Analysis (PRISMA-P) 2015 statement [28]. Qualitative and quantitative studies published until February 2020, conducted in Ethiopia, written in English were searched extensively systematically in the following databases and search engines: PubMed, Cochrane Library, POPLINE, Google Scholar, HINARI, African Journals Online (AJOL), and WHO Global Health Library. In addition, a manual review of the references from eligible included studies was carried out via back and forth searches.

The search strategy and engine were developed using keywords/free text terms and Medical subject headings (MeSH) terms in various combinations for the following concepts: quality of intrapartum care, disrespect or abuse, mistreatment or obstetric violence, attitude of health personnel, or professional misconduct, childbirth, labor and delivery, and Ethiopia.

Search strategies employed in PubMed was (((disrespectful) AND abuse) AND “Delivery, Obstetric”[Mesh]) AND “Ethiopia”[Mesh].

### 2.2. Inclusion Criteria

Quantitative and qualitative studies of primary data were conducted in Ethiopia, reports on indicators that can be categorized under disrespect and abuse of women during childbirth, reports on contributing factors, consequences of disrespect and abuse of women during childbirth, reports dealt with quality of care related to the disrespect and abuse of women during childbirth, explore actual experiences and perception of women and their companions during childbirth and reported any form of disrespect and abuse, and reports on health care providers perspective on disrespect and abuse were included.

### 2.3. Exclusion Criteria

Studies done out of Ethiopia, which were unable to access the full text, and studies published in other languages other than English were removed.

The primary outcome of this review was to determine the nature/forms and causes of disrespect and abuse of women during childbirth in Ethiopia, and the secondary objective was to identify contributing factors and consequences of disrespect and abuse of women during childbirth. According to Bowser and Hill framework and classification [24], disrespectful and abusive behavior during childbirth is an act of the following: physical abuse, nonconfidential care, nonconsented care, nondignified care, abandonment of care, and discrimination and detention in the facilities during childbirth.

### 2.4. Data Extraction

Initially, an advanced and systematic search was made via the identified databases listed. In addition, a manual review of the references of the included studies was undertaken to access additional relevant articles. Next, studies published other than the English language, conducted out of Ethiopia, unrelated and irrelevant articles based on their title and abstract were excluded. Then, those remaining articles were imported to Endnote version 8, and duplicate articles were removed. Data was extracted by using the Joanna Briggs institute structured data extraction format. Presented using a table consisting of the following items: first author's name and year of publication, study location, study design, study description, sample size, type and characteristics of disrespect and abuse, analysis method, results, and contributing factors.

### 2.5. Quality Appraisal

The quality assessment of studies for the quantitative cross-sectional was assessed using the Centre for Evidence-Based Management (CEBM) Survey Critical Appraisal Tool [29], and qualitative studies were assessed using the Critical Appraisal Skills Program (CASP) tool [30]. The qualities of included studies were rated as low, medium, or high. All the studies were kept for the final analysis.

### 2.6. Strategy for Data Synthesis

We adopt Bowser and Hill framework for data synthesis. This typology was used because, most of the included articles, their method of data synthesis was according to this framework. To fulfill the objective of this review, this framework was found to be suitable. According to the framework, disrespect and abusive care are categorized into seven domains and categorizes contributing factors into individual and community level, policy and governance level, providers, and service delivery factors. The findings were grouped, interpreted, and summarized in these categories. For quantitative synthesis, we documented the type and form of disrespect and abuse experienced in each category, contributing factors, and consequences in the form of percentages. For qualitative synthesis, we put forward statements and quotes from the experience of women companions and caregivers. The protocol of this systematic review is under the registration process with the prospective registration number of 175547 for systematic reviews (PROSPERO acknowledgment of receipt [175547]).

## 3. Results

### 3.1. Study Selection and Characteristics

**Figure 1:**
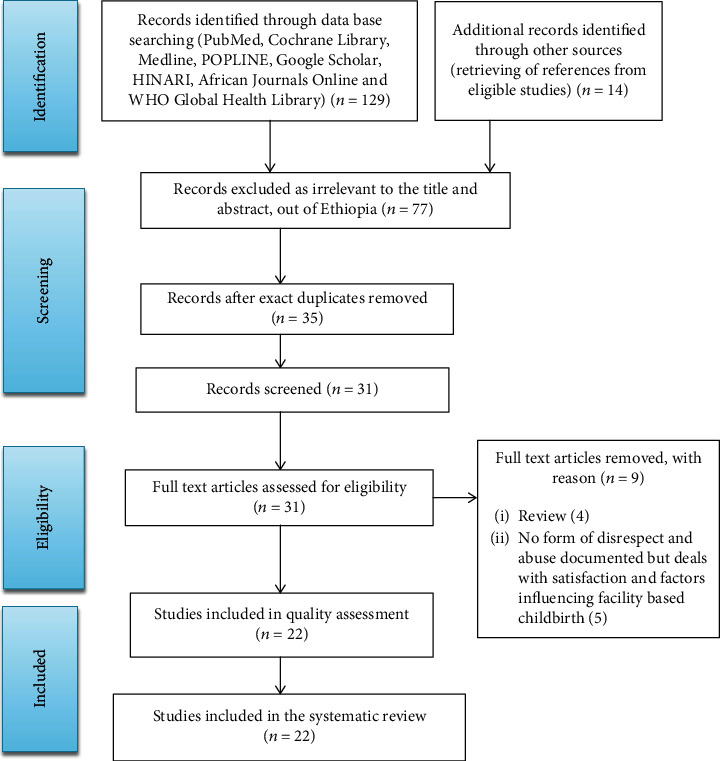
PRISMA statement presentation for systematic review of women's and health care providers' perspectives on disrespect and abuse during childbirth in Ethiopia.

The online search yielded 143 citations through database searching and retrieving of references from eligible studies. Out of this scan, 121 retrieved studies were omitted via a step-by-step procedure due to the following reasons: 77 excluded as irrelevant by title and abstract, 35 removed as duplicates, 4 of them were review, and 5 forms of disrespect and abuse were not documented (Figure 1).

Of the 22 studies included in this review, 16 of them were quantitative cross-sectional studies, 5 qualitative studies, and 1 was mixed-method study. Of the 16 quantitative cross-sectional studies included, two were direct observational studies [31, 32]; and three of them were perspectives of health care providers' [33–35]. Nine of the included studies were performed simultaneously in both rural-urban settings [31, 35–42], eleven in urban setting [14, 33, 34, 43–51] and one in rural setting [32]. Three of the studies were conducted in the capital (Addis Ababa) [33, 43, 46], two in Tigray region [42, 47], four in Amhara region [14, 34, 44, 49], five in Oromia region [36, 37, 39, 50, 51], three in SNNP region [38, 40, 45], two in both SNNP and Amhara regions [32, 35], one in four of the regions (Tigray, Oromia, SNNP and Amhara) [41], and one was done nationwide of rural settings [31] (Table 1).

### 3.2. Synthesized Outcomes

Prevalence studies in Ethiopia have shown prevalence reports revealed that women experience at least one form of disrespect and abuse during childbirth ranging from 21.1% [32] to 98.9% [45], which are unacceptably high levels of obstetric violence and mistreatment care.

Our synthesized findings were recorded using Bowser and Hill framework [24] and categorized under the following domains.

#### 3.2.1. Physical Abuse

Physical abuse during childbirth was reported in eighteen of the cross-sectional and qualitative studies [14, 31–37, 39–45, 49–51] and ranged from 9% to 87.9%. To include, being beaten, slapped, and/or pinched by the health care provider [14, 31, 33–37, 39, 41, 44, 45, 48–51] and care providers used force to push the abdomen down (used fundal pressure) to deliver their babies [32, 40, 45], episiotomy done or perineum sutured without antipain (anesthesia) [39, 43, 45], restrained or tied down during labor [36, 40], legs apart harshly and forcefully during labor were reported in 33.8% [36] and 13.9% [50] of them, 1.8% faced previous experience of sexual abuse by the caregiver [37], and a qualitative studies have argued that painful per-vaginal exam have been carried out multiple times without notifying the findings [34, 42]; “The caregivers have psychologically wounded us, They have come and did vaginal exams repeatedly as easy as anything but it is a huge trauma to us” [42].

#### 3.2.2. Nonconsented Care

The women's right to information and informed consent was the most consistently dishonored right during facility-based childbirth, as recorded by between 16 to 92.5 percent of women in fifteen of the studies [32, 34–36, 39–41, 43–47, 49–51]. A significant number of women reported caregivers did not introduce their name during admission time [43–45, 50, 51], caregivers did not tell the progress and consecutive evaluations to the women during labor [41, 45], unconsented episiotomy and per-vaginal examination [32, 34, 36, 45], unconsented cesarean delivery, instrumental delivery, and labor augmentation [35, 36, 39, 45], and written and verbal informed consent was not taken before/while any procedure [39–41, 43, 44, 47, 49]. A woman delivered at the health center responded, “He used a metal instrument to take the baby out and told me to react when I have contractions, he did not ask my permission, or informed me of what he is doing” [35].

#### 3.2.3. Nondignified Care

With exception of the overt forms of abuse, women's description and perception of nondignified care are context-specific. According to our context, the women's right to be treated with dignity and respect was violated during childbirth, as stated by a difference of 8 to 55.3 percent of women in all of the included studies [14, 31–51]. Women experience insult, shout, intimidation, and threaten without provocation and insensitiveness to the patients [14, 33–36, 39–41, 44–47, 50, 51]. In a qualitative study in the migrant community, one mother said, “In the past they were yelling at us, they insult labouring women for not being clean. They were saying that why you did not shower, you have bad smell. To avoid this humiliation we were giving birth at home. But now it has been changing” [46]. They made negative comments and mocking [32, 34, 44] …“Some of them heap scorn on you when you are in labor” [34]. Unfriendly, unwelcomed, and impolite approaches of health care providers were found in seven of cross-sectional studies [14, 32, 38, 43, 45, 48, 51]. In a qualitative study done on women who gave birth at home and who had previous experience of facility-based childbirth, one woman stated, “The traditional birth attendants will take care of your feelings; they treat you with sympathy. They are well aware of and concerned about our culture, so they never do something that can disappoint you. But those in health facilities act as if they were from another planet. They enjoy your pain and degrade you from humanity. I don't even understand why they are here if they don't respect and serve the needy” [38]. Women confirmed that their parents were not permitted to accompany them during labor [38, 41, 45].

#### 3.2.4. Nonconfidential Care

The frequency of nonconfidential care that reported on prevalence ranges from 11% to 81.7%.

Women's right to privacy and confidentiality violation was reported in sixteen of the studies including lack of physical privacy (curtains, screening, and any visual barriers) [31, 34–36, 39, 41, 44, 45, 50, 51], lack of auditory privacy (discuss sensitive health information publicly and overheard by others) [32, 36, 40, 44, 47, 50], and medical history disclosed without consent in 7.2% of them [36]. In the qualitative study done on patients and care providers perspective, one of the participants stated, “I told the midwife not to allow [students to enter and observe care], but they were already in the room on practical learning, and the midwife didn't want to send them out once they were in. In the future, I don't want that” [34]…one trainee student also stated, “We students were many in number, and clients got ashamed to be free in front of us [to permit vaginal exams], and did not comply with the orders given by providers” [34].

#### 3.2.5. Discrimination

In Ethiopia, 2.2% to 54.6% of women, their right to equally, discrimination-free and equitable care was violated on basis of circumstances by caregivers. Women reported discriminatory care in eight cross-sectional studies, to include because of their traditional belief [45], being rural residence [36, 45], 12.5% due to low educational status or no formal education [45], race/religion or ethnicity [40, 44, 51], low economic status [36, 40, 44], being teenager (young age) [36, 40, 45], 5.4% discriminated because of serostatus positive for HIV [40], and 13.4% did not get equitable care because of difficulty in language [51]. One woman from rural residence stated the level of discrimination as, “By the time I went there in labour, there was one woman who came from the urban area, she was there before me but I gave birth before her. They were taking care of her and treating her better than they did for me ... they visited her more frequently, and they comfort her than me” [35].

#### 3.2.6. Abandonment/Neglect

A total of 4.3 to 53.8% of women in 14 surveys, including left alone or unattended during labor and delivery, reported denial of women right to healthcare and to the highest attainable level of health and continuous care [31, 33, 34, 40, 43–45, 47, 49, 51], pain management neglected despite they need it [14, 42], did not come on need [34, 43, 44, 50, 51], movement limited for a long time [47], and 16.5% of women reported delayed procedure after decision [40].

#### 3.2.7. Detention in Facilities

Detention at health facilities was reported by a range of 2.9% to 25.9% of women in four studies for the issue of failure to pay and against their will [36, 44, 51] and detained for fear of home delivery [42].

### 3.3. Factors Contributing to Disrespect and Abuse during Childbirth

In this review, the factors leading to disrespect and abuse during childbirth in Ethiopia included research from the experience and interpretation of women, the experience of care providers, trainees, and discussed in their results.

#### 3.3.1. Individual and Community-Level Factors

*(1)Normalization of Disrespect and Abuse during Childbirth*. Asefa et al. [33] and Ukke et al. [45] discussed in their finding as the widespread observation and experience of disrespect and abuse indicates, it is normalized, culture and attitudinal among women and caregivers in health institutions. For a majority of women who are perceived disrespect and abused, while reported as not disrespected and abused, it means disrespectful and abuse care well-thought-out to be not thoughtful by service users and tolerable community [32, 39, 43]. A resident dweller woman who had not received formal education interrogated as, “...Yes, because they are doing this to save my life, I don't mind ... Yes it is for my benefit and it was not meant to hurt me” [35].

*(2)Lack of Autonomy and Empowerment*. Four studies found that those mothers residing in rural had low educational status, and low socioeconomic status is more likely to face disrespected and abused care [14, 35, 44, 46, 49]. The authors of those studies link the factors with disrespect and abuse care as; with regard to low socioeconomic status, those mothers who had control over finance may have a high level of utilization of institutional delivery, and this would have a lot more exposure to the care system and aware their rights. Related to educational status, educated women are better aware of their rights and developed self-confidence decreases the disparity in power between caregiver and service users, making them less affected by disrespected and abused care. However, Mihret 2019 [49] did not agree with these postulations. While less informed and rural residents are more likely to face disrespect and abusive care, as rural residents are not aware of their rights and may never have been introduced to the care system by the abusive and bad approach of caregivers to view it as status quo and did not disclose it as disrespectful and abusive care. With regard to education, Mihret 2019 [49] concluded that perhaps educated women are more conscious of their rights and are thus more open to disclosing incidents of disrespect and abusive behaviors.

#### 3.3.2. National Laws and Policies, Human Rights, and Ethics

*(1)Lack of Enforcement of National Laws and Policies and Lack of Legal Redress Mechanisms, Supervision*. The lack of redress mechanism in case of complaints was reported by 65.2% of respondents as a constraint for respectful maternity care [40]. Sheferaw et al. [31] also discussed the importance of the SBM-R quality improvement approach; facilities that adopted the SBM-R quality improvement approach demonstrated a higher level of Respectful Maternity Care compared to those who did not.

#### 3.3.3. Providers and Service Delivery

*(1)Provider Distancing as a Result of Training*. None of the included studies provide an evidence for this factor as a contributor. However, Sheferaw et al. 2017 [31] speculated on their discussion session as, lack of preservice, in-service training/education for the maternal health work force about respectful maternity care contributes to the harsh relationship of service users and providers.

*(2)Provider Demoralization Related to Weak Health Systems, Shortages of Human Resources, and Professional Development Opportunities*. Poor support is from facility management to the unreserved effort of provider [33, 40, 45], inconvenient working environment (low satisfaction, low payment relative to their effort) [33, 34, 38], and staff shortage [34, 51].

High workload resulted from high patient flow in tertiary hospitals and duty night shift made them overworked, tired, and easily irritated [32, 33, 41, 45, 51], lack of resource, and failing infrastructure [32, 34, 39, 40, 42, 51]. From a qualitative evidence, one urban woman gave birth for the first time quoted as follows, “I was left alone on a couch for 7 hours with instruction to remain in the same position, which was inconvenient and very cold without any beddings. Then after …they forced me stand up and move…I was not strong enough yet. What annoyed me most was they made me collect my blood stained linens and clothes” [42].

### 3.4. Consequences

Many evidences have showed that there is a proven correlation between disrespectful and abusive facility-based childbirth care and not to the use of facility-based childbirth services currently or in the future [13, 24]. As a result, the following consequences have been reported from the included articles reviewed.

### 3.5. Nonutilization or Delayed Utilization of Skilled Delivery Services

The bad approach of health care providers strangulated by lack of legal redress mechanisms and supervisions dictates women to restrain from using skilled birth services, prefer to deliver at home with traditional birth attendants, no intention to come health institutions in the coming future, influence women's decision about how, when and with whom to give birth [14, 33, 36, 38, 44–46].

### 3.6. Psychological Consequences

Violation of human rights has led to psychological humiliations, and this creates social and psychological detachment between care providers and service users [32, 39, 42, 43]. 12 percent of women reported they experiencing depression in the last 12 months of their childbirth [37].

## 4. Discussion

This systematic review found that disrespectful and abusive care of women during childbirth is repeatedly practiced care in Ethiopia. The nature and forms of this practice varied from study to study; the most repeatedly dishonored right during facility-based childbirth in Ethiopia was nondignified care, which is reported in all of the included studies, in addition to this, physical abuse has been stated frequently and comparatively that the least commonly reported abuse was detention in health facilities without their will, listed only in four of the included studies.

With the exception of overt forms of abuse, most of the descriptions and perceptions of women are context-specific; and this can be subjected to under-or-over reporting, as this behavior may be accepted as normal for some women and may be considered to be abuse or disrespect by some women. This poses a difficulty in assessing precisely the incidence of disrespect and abusive care. To avoid this confusion, this review tried to provide the combination of quantitative, qualitative, mixed, observational studies, and not only women's experience but also companions opinion, and caregivers experiences to D and A.

Physical abuse, nondignified care, nonconfidentiality care, nonconsented care, discrimination, abandonment/neglect, and detention in health facility identified by the findings of this review have been widely recorded in many settings as a significant obstacle to the utilization of facility-based childbirth services [2, 6, 8, 11, 12, 27, 52, 53]. In line with the findings of this review, normalization of D and A care, lack of autonomy and disempowerment, lack of redress mechanism and supervision for complaints and violations, lack of resources and failing infrastructure, and staff shortages have been described as key contributors to D and A [2, 8, 11, 12].

While negative experience and unsatisfactory events of women have been recorded in many situations, positive experiences and the development of a homely environment have been reported in some settings (e.g., preparing coffee ceremony in labor ward) [46] and other settings should take lessons and share these positive experiences. In contrast, in some settings, health care providers have forced women to deliver in ill-prepared health facilities by intimidating them with punishments such as withholding social activities, participating in ceremonies, and restricting them to job opportunities instead of maintaining a positive and homely environment [42].

The country's health system affects respectful and nonabusive care of women during childbirth by endangering the working conditions of the primary care providers, making too few workers but many service users, less timely care, failing facilities, and shortage of resources, failing to upgrade and improve personnel development of staffs [54–56]. These ill-structured health systems generate long waiting time, lack of timely care, unfriendly care, negligence, lack of physical and auditory privacy, and overcrowded due to high patient flow. In addition, this encourages women to view and assess as incompetence on the part of providers only, possibly due to heavy workload, inconvenient working environment, and poor satisfaction due to low pay.

This review also examined that not only to confirm that Ethiopia is experiencing a similar situation but also to investigate several accounts of previous experience of sexual abuse by caregivers over their lifetimes. This bad experience prevents women from attending health facilities to pursue maternal health services. This review also illustrated that the majority of D and A victim groups are minorities (pregnant teenagers), low socioeconomic status, low educational status, patients living with HIV, and who had language difficulties. Notwithstanding their status, women have the right to obtain equal treatment, free from discrimination, and equitable care during childbirth [1] [1]; however, in Ethiopia they are not equal to those rights on the ground. The impact of these bad experiences has led many Ethiopia women to choose traditional birth attendants than skilled birth attendants; believed that the former regards you with sympathy, concerns with culture and still treats them with irrespective of their status [57].

The introduction of standard-based management and recognition (SBM-R) approach to improving the quality of maternal health services in some institutions has shown a higher degree of Respectful Maternity Care relative to those who have not adopted the SBM-R quality improvement approach [31]. However, this approach is confined to some institutions, and its extension to other centers requires a great deal of effort. Besides this inadequate performance of this approach, lack of legal redresses mechanism in case of complaints in institutions has made it difficult to promote respectful maternity care.

The Government of Ethiopia has implemented a compassionate and respectful care initiative in its five-year Health Sector Transformation Plan (HSTP) to resolve this startling issue [19]. This initiative focused mainly on equity and quality of care delivery, with a particular emphasis on maternal health services, through offering training for pre and in-service health practitioners in the area of interpersonal and communication skills [58]. Respectful maternity care is also included in Basic and Emergency Obstetric Care (BEmONC) of training session focused on increasing of awareness and culturally responsive care during childbirth, educating women on what to expect during childbirth including their right to informed consent, privacy, and confidentiality, and requesting their preferences during childbirth [59].

While such measures are in effect, policies to promote Respectful Maternity Care as a standard of practice are seldom adopted, only value of paper and for the sake of political consumption.

## 5. Limitations and Strengths

Considering the limitation of the study, the findings should be interpreted. We followed the Bowser and Hill landscape analysis on disrespect and abuse to inform the evidence synthesis to achieve the objectives of the review. We have tried to include few studies on the experiences of provider in order to recognize the contributing factors and suggesting solutions, however, the included studies are not enough to enrich with more information due to limited studies. We also integrate research underscore on the ideas of these women's companions during childbirth, since women might not be in a strong place to articulate their experiences at times during labor and delivery or immediately after childbirth, in which case the observation of companions was of immense benefit. Despite the low quality of the articles, all of the studies meet the inclusion criteria were included; this may introduce the issue of small sample size, risk of selection, recall, and courtesy bias. Another drawback would be that, though we included unpublished papers, still there might be remaining of unpublished articles concerning this topic. While research conducted in Ethiopia, published in languages other than English, has not been found, the collection of papers published in English only may introduce a bias.

## 6. Conclusion

This systematic review renowned that disrespectful and abusive care of women during childbirth is repeatedly practiced care in Ethiopia, and this finding specifically positions the contributing factors in a broad range and consequences of D and A as a deterrent for utilization of facility-based childbirth at present and subsequent times. To address this alarming problem, therefore, participation in empowering and educating of women on their rights and expectations during childbirth, creating conducive environments for health care providers, strengthening of health systems on respectful maternity care, providing training for pre and in-service care providers on interpersonal and communication skills, implementation of standard-based management and recognition (SBM-R) approach to improve the quality of maternal health, strong legal redresses mechanism in case of complaints, and implementing of policies for standard care of respectful maternity care is utmost importance. In addition, observational, qualitative, and mixed types of studies are required to provide comprehensive evidences on disrespect and abuse during childbirth in Ethiopia.

## Figures and Tables

**Table 1:** Summary results of included studies.

Author, year	Study setting and location	Study design and description	Sample size	Type and characteristics of D and A	Results	Type of analysis done	Contributing factors
Asefa et al., 2018 [33]	Addis Ababa, urban	Cross-sectional (interviewer-administered questionnaire)	57 health professionals from the health center and hospital (convenience sampling)	Slapping, hitting, unattended labor/left alone, privacy not protected, detaining at health facility	25.9% reported physical abuse, 34.5% privacy was not protected, 13.2% observed unattended labor	Survey data analyzed by tabulations	Normalization of D&A as a culture, high workload, poor support from facility management, discomfort of the working environment
Wassihun and Zeleke 2018 [14]	Bahir Dar, urban	Cross-sectional (interviewer-administered questionnaire)	Survey of 284 mothers who gave birth selected randomly	Did not support pain relief, slapped, insult, treated not friendly way	30.3% with no support in pain relief, 34.5% slapped by, 31.3% insulted, 68.3% of them treated in unfriendly approach	Survey data analyzed by tabulations and logistic regression	Low family monthly income
Ukke et al., 2019 [45]	Arba Minch, urban	Cross-sectional (interviewer-administered questionnaire)	Survey of 281 postnatal mothers selected systematically	Nondignified care, doing episiotomy without anesthesia, nonconsented care, discriminated care, abandonment of care	18.8% sutured their perineum without anesthesia,36.7% nondignified care, 92.5% of nonconsented care, 18.1% perceived discriminatory care	Survey data analyzed by tabulations	Normalization, lack of support from management body, workload and dissatisfaction by the hospital staffs, low socioeconomic status
Wassihun et al., 2018 [44]	Bahir Dar, urban	Cross-sectional (interviewer-administered questionnaire)	Survey of 422 households selected by systematic random sampling	Physical force, insult, nonconsented care, physical privacy not respected, personal info discussed with others	23.2% reported physical force, 27.1% insult, 25.1% report did not obtain any consent, 8.3% reported did not use barriers for privacy, 5.4% claimed their personnel info was discussed	Survey data analyzed by tabulations and bivariate and multivariate for associations	Low maternal family income
Banks et al., 2017 [32]	SNNP and AMHARA, rural	Quantitative cross-sectional direct observation (semistructured checklist)	Survey of 204 mothers who gave birth and client-provider interaction was observed in 193 of them selected randomly	Fundal pressure, lack of consent, auditory privacy not respected, physical privacy not respected	14.1% of them fundal pressure was applied, 68.4% of them lack of consent, 21.1% of them auditory privacy was not respected, 20.7% physical privacy was not provided	Survey data analyzed by tabulation and fishers exact test	Normalization of behavior and circumstances by both woman and provider, high patient flow, failing infrastructure
Bobo et al., 2019 [36]	West Oromia, urban-rural	Cross-sectional (interviewer-administered questionnaire)	807 surveys of mothers who gave birth in the institution, systematic random	Harshly forcing leg apart, shouted at, unconsented episiotomy, and C-section, discriminate on basis of status	33.8% legs apart harshly, 31.4% report shouting at, in 48.9% of them unconsented episiotomy was done; in 39% of them, physical privacy was not provided.	Survey data analyzed by tabulations and bivariate and multivariate for associations	Experience with the health facility setting, lack of a companion throughout labor and delivery services
Siraj et al., 2019 [51]	Jimma, urban	Cross-sectional (interviewer-administered questionnaire)	290 surveys of immediate postnatal mothers (convenient consecutive sampling)	The right to information not protected, ill-treatment, confidentiality and privacy not protected, detention	90% reported the right to info denied, 87.9% report physical harm and ill-treatment, 25.9% report detention	Survey data analyzed by tabulation	High patient flow and faced significant resource and staff shortage
Mekonnen et al., 2019 [37]	Bale, urban-rural	Cross-sectional (interviewer-administered questionnaire)	565 surveys of immediate postnatal mothers, multistage then SRS	Verbal abuse and physical threaten	11% verbally threatened, 17.3% physically abuse	Survey data analyzed by tabulation and chi-square	Not specified
Warren et al., 2017 [52]	Hadya zone, urban-rural	Qualitative (focused group discussion and in-depth interview), semistructured checklist	8 FGD (8 to 10 members per group) and 16 key informant interviews (purposive sampling)	Bad approach, treated neglect and arrogantly, not involvement of companionship	Care providers did not show any empathy, they did not tell what is going on, physical privacy was disgraced in front many people, insulting	Thematic content analysis	Low degree of job satisfaction, routine, and attitudinal
Mirkuzie, 2014 [46]	Addis Ababa, urban	Qualitative (in-depth interview), semistructured checklist	11 women who gave birth at home in IDI and 28 in FGD	Shouting at, not treat them in a respectful way, insulting, misinformation	Yelling at and did not have any empathy, not treating in a women-friendly way, run out of resources, misinformation, and misdiagnosis that creates hesitation	Framework and content analysis	Pregnant migrant women often have low education and low economy
Burrowes et al., 2107 [34]	Debre Markos, urban	Qualitative (in-depth interview and FGD), semistructured checklist	23 women participated in FGD and 19 (4 health care provider and 15 students) in IDI, purposive	Autonomy not respected, verbal abuse, physical abuse, nonconsented care, abandonment	Exacerbation of pain by shouting and insulting, pinching and slapping clients to open up their leg, restriction of fluids and food, deny to adopt preferred position	Framework and content analysis	Shortages of personnel, lack of supplies, heavy workload, low payment compared to effort
Tefera and Abeya, 2019 [39]	Bishoftu, semiurban	Mixed (cross-sectional study and FGD), interviewer-administered questionnaire	351 surveys of pregnant women selected systematically and 6 FGD (8 to 10) selected purposively	Physical harm, prohibition of mothers informed consent, nonconfidential care, abandonment	18.5% of report episiotomy was done without anesthesia and suturing without antipain, 27.4% reported prohibition of informed consent, not using of screening, scolding and hitting	Survey data analyzed by tabulation and thematic analysis	Lack of infrastructure, normalization, lack of good attitude of health care provider
Getachew, 2019 [40]	Hossana, urban-rural	Cross-sectional (interviewer-administered questionnaire)	577 surveys of mothers selected by systematic random sampling	Neglect and abandon, ineffective communication, loss of autonomy, lack of supportive, discrimination	44.9% reported lack of informed consent, 21.2% report discussion overheard by others, 20% neglected and abandoned, 5.4% discriminated by status	Survey data analyzed by tabulation	Lack of resource, lack of policy, facility culture, lack of redress mechanisms,
Sheferaw et al., 2019 [41]	Oromia, SNNP, Amhara and Tigray, both urban and rural	Cross-sectional (interviewer-administered questionnaire)	379 surveys of postnatal mothers of selected systematically	Lack of autonomy, poor perception, not responding to questions, no explanation during labor, not allowing companion	Poor rapport between patient and provider was reported in 72% of them, 28% report not allowing birth companion, 56% did not adopt the preferred choice of position during labor	Survey data analyzed by tabulation and bivariate and multivariate for association	High patient volume and workload
Gebremichael et al., 2018 [47]	Tigray, urban	Cross-sectional (interviewer-administered questionnaire)	Survey of 1125 of women who gave birth within the preceding year selected by systematically	Shouted at, scolded/insulted, discouraging/become negative, restriction of movement	12.5% shouted at, 10.5% scolded/insulted, 5% claimed care provider as discouraging,, movement during labor was restricted in 3.8% of them	Analyzed by tabulation and negative binomial and multivariate for association	Not specified
Mihret, 2019 [49]	North West Amhara, urban	Cross-sectional (interviewer-administered questionnaire)	Survey of 409 postnatal mothers selected by systematic	Physical abuse, nonconsented care, nonconfidential care, discriminatory care, neglected care	49.6% experience physical abuse, 63.6% reported nonconsented care, 32.3% report nonconfidential care	Analyzed by tabulation and negative binomial and multivariate for association	Low educational status and residing rural
Sheferaw et al., 2017 [31]	Nationwide, urban and rural	Cross-sectional direct observation(semistructured checklist)	240 of women during labor and delivery were observed	Physical abuse, verbal abuse, privacy violated, abandonment/left alone	9% experience physical abuse, 8% experience mistreatment of verbal abuse, in 17% of them, privacy was violated and 19% of the mothers were left alone	Chi-square, bivariate and multivariate, tabulation	Insufficient in-service training and preservice education, unavailability of redress mechanism,
Bante et al., 2020 [48]	Harar, urban	Cross-sectional (interviewer-administered questionnaire)	Survey of 425 women nominated by systematic random sampling method	Untimed care, unfriendly care, abused care, discriminatory care	40.2% claimed that the care was unfriendly, 42.1% mothers experience abuse care either physically or verbally, 54.6% of them respond the care was discriminatory on status	Survey data analyzed by tabulation	Congestion of patient with less concentrating of service delivery
Asefa and Bekele, 2015 [43]	Addis Ababa, urban	Cross-sectional (interviewer-administered questionnaire)	Survey of 173 women nominated by systematic random sampling	Did not receive pain relief on request, uncaring in a culturally acceptable way, did not obtain consent	23.7% of them did not receive pain relief upon request, 9.2% of them did not demonstrate culturally acceptable way, 48% did not obtain informed consent	Survey data analyzed by tabulation	Facilities being as teaching hospital, normalization of care
Gebremichael et al., 2017 [42]	Tigray, both urban and rural	Qualitative (focused group discussion) (semistructured checklist)	8 FGD (8 to 10 members per group) which accompanies A total of 62 women who gave birth in the year prior to the study	Did not explain the findings, detention without their will, lack of support for basic physiologic needs	Perform PV multiple times but did not explain the findings, women complain about detention at the health facility for the fear of home delivery, pain was not managed	Thematic and content analysis	Negligent and incompetent providers, scarcity of resources
Molla et al. 2017 [35]	SNNP and Amhara, urban and rural	Qualitative (IDI and FGD), semistructured discussion checklist	IDI with 4 midwives and 42 women gave birth at home and institutions, 8 FGD with 63 family members accompanying labor women	Insulting, pushing/hitting, consent not obtained, discrimination based on residence, no physical privacy	Yelled when out of bed, slapping in denying, clenching of legs apart, doing instrumental delivery without consent, differentials in care for urban and rural women	Thematic and content analysis	Disempowerment and loss of autonomy, normalization
Bekele et al., 2020 [50]	Oromia, urban	Cross-sectional (interviewer-administered semistructured questionnaire)	Survey of 321 women selected by systematic random sampling	Forcing leg apart, physical and auditory privacy not respected, did not introduce, shouting, ignored when needed	13.9% report legs harshly apart, 33.5% physical privacy disgraced, 72.2% did not introduce themselves, 13.3% ignored when the care is needed	Survey data analyzed by tabulation	Not specified

## Data Availability

The datasets used and/or analyzed during this study are available within the manuscript and/or additional supporting files.

## Abbreviations

D and A: Disrespect and abuse
SNNP: Southern nation nationality people
FGD: Focused group discussion
IDI: In-depth interview.
